# Resistance exercise training and in vitro skeletal muscle oxidative capacity in older adults

**DOI:** 10.14814/phy2.12849

**Published:** 2016-07-12

**Authors:** Kyle D. Flack, Brenda M. Davy, Martin DeBerardinis, Nabil E. Boutagy, Ryan P. McMillan, Matthew W. Hulver, Madlyn I. Frisard, Angela S. Anderson, Jyoti Savla, Kevin P. Davy

**Affiliations:** ^1^ United Stated Department of AgricultureAgricultural Research ServiceGrand Forks Human Nutrition Research CenterGrand ForksNorth Dakota; ^2^ Department of Human Nutrition, Foods, and ExerciseVirginia TechBlacksburgVirginia; ^3^ Fralin Translational Obesity Research CenterVirginia TechBlacksburgVirginia; ^4^ Center for GerontologyVirginia TechBlacksburgVirginia; ^5^ Department of Internal MedicineSection of Cardiovascular MedicineYale University School of MedicineNew HavenConnecticut; ^6^ Metabolic Phenotyping CoreVirginia TechBlacksburgVirginia; ^7^ Department of BiologyPikes Peak Community CollegeColorado SpringsColorado; ^8^ Department of Human DevelopmentVirginia TechBlacksburgVirginia

**Keywords:** Metabolism, mitochondria, oxidative damage, strength

## Abstract

Whether resistance exercise training (RET) improves skeletal muscle substrate oxidative capacity and reduces mitochondrial production of reactive oxygen species in older adults remains unclear. To address this, 19 older males (≥60 years) were randomized to a RET (*n* = 11) or to a waitlist control group (*n* = 8) that remained sedentary for 12 weeks. RET was comprised of three upper body and four lower body movements on resistance machines. One set of 8–12 repetitions to failure of each movement was performed on three nonconsecutive days/week. Improvements in chest press and leg press strength were assessed using a three‐repetition maximum (3 RM). Body composition was assessed via dual energy X‐ray absorptiometry. Muscle biopsies were obtained from the vastus lateralis muscle at baseline and at both 3 weeks and 12 weeks. Palmitate and pyruvate oxidation rates were measured from the ^14^ CO_2_ produced from [1‐^14^C] palmitic acid and [U‐^14^C] pyruvate, respectively, during incubation of muscle homogenates. PGC‐1*α*, TFAM, and PPAR *δ* levels were quantified using qRT‐PCR. Citrate synthase (CS) and *β*‐HAD activities were determined spectrophotometrically. Mitochondrial production of reactive oxygen species (ROS) were assessed using the Amplex Red Hydrogen Peroxide/Peroxidase assay. There were no significant changes in body weight or body composition following the intervention. Chest press and leg press strength (3RM) increased ~34% (both *P* < 0.01) with RET. There were no significant changes in pyruvate or fatty acid oxidation or in the expression of target genes with the intervention. There was a modest increase (*P* < 0.05) in *β* HAD activity with RET at 12 weeks but the change in CS enzyme activity was not significant. In addition, there were no significant changes in ROS production in either group following RET. Taken together, the findings of this study suggest that 12 weeks of low volume RET does not increase skeletal muscle oxidative capacity or reduce ROS production in older adults.

## Introduction

Advancing age is characterized by a progressive decline in skeletal muscle mass, termed sarcopenia, that results in significant disability, morbidity, and mortality (Berger and Doherty 2010; Doherty 2003; Hirani et al. 2015). Skeletal muscle mitochondrial function declines and oxidative stress increases with advancing age (Petersen et al. 2015; Short et al. 2005) and these changes have been implicated in the etiology of sarcopenia (Cadenas and Davies 2000; Joseph et al. 2015). Oxidative damage to macromolecules by reactive oxygen species (ROS), particularly within mitochondria, can accelerate mitochondrial dysfunction and further ROS production leading to a vicious cycle that contributes to apoptosis, muscle loss, and sarcopenia (Cadenas and Davies 2000; Joseph et al. 2015). Because the population of US adults over 65 years of age is expected to double in the next four decades (Olshansky et al. 2009), age‐related loss of muscle will become an even greater public health problem. Therefore, improving our understanding of the strategies that may be effective in mitigating age‐related changes in skeletal muscle will be critical to thwarting the public health consequences related to these demographic changes.

Resistance exercise training is a safe and effective strategy for increasing muscle mass and strength in older adults (Johnston et al. 2008; Tarnopolsky 2009). However, whether RET increases skeletal muscle oxidative capacity in older adults remains unclear. The results of prior studies suggest that RET is not associated with increases citrate synthase (CS) activity, a commonly used biochemical marker of mitochondrial density and skeletal muscle oxidative capacity (Blomstrand et al. 1997; Larsen et al. 2012), in older adults (Parise et al. 2005a,b). However, Melov et al. (2007) reported a global enrichment of genes associated with mitochondrial function in skeletal muscle following RET in older adults. To our knowledge, it is not known whether RET increases oxidation of substrates in skeletal muscle. Therefore, the purpose of this study was to test the hypothesis that RET would increase skeletal muscle oxidative capacity. In particular, we hypothesized that RET would increase the expression of target genes of key transcriptional regulators of mitochondrial biogenesis and metabolism, CS and *β*‐hydroxyacyl‐coenzyme A dehydrogenase (*β*HAD) activity, a key enzyme involved in beta oxidation of fatty acids to acetyl CoA, and in vitro skeletal muscle fatty acid and pyruvate oxidation. In addition, increases in antioxidant enzyme activity (Parise et al. 2005a,b) and reduced lipid peroxidation (Vincent et al. 2002) and oxidative damage to DNA (Parise et al. 2005a) have been observed following RET in older adults. However, whether RET reduces the mitochondrial production of reactive oxygen species remains unknown. Thus, this study was designed to also address this issue.

## Materials and Methods

### Participants

Twenty nonobese (body mass index <30 kg/m^2^) older (≥60 years) males volunteered to participate in this study. Only men were included in this initial investigation to avoid the potential gender differences in muscle adaptations to RET in older adults (Bamman et al. 2003). All participants were free of overt chronic diseases by medical history and had obtained medical clearance from their personal physician prior to beginning the study. One participant was excluded due to initiation of steroid treatment after enrollment. Four individuals in the resistance exercise intervention were taking a statin (3 on Atorvastatin, 1 on simvastatin; 20–40 mg OD) for at least a year without muscular complaints. All participants were sedentary to recreationally active and were free of orthopedic conditions that would preclude engaging in a resistance exercise intervention. Participants taking over the counter supplements, multi‐vitamins, or aspirin completed a two‐week wash‐out period prior to the first biopsy. The nature, purpose, risks, and benefits were explained to each subject before obtaining informed consent. The experimental protocols were approved by the Virginia Tech Institutional Review Board.

### Intervention

Subjects were randomized to 12‐week intervention consisting of supervised resistance exercise on three nonconsecutive days per week using machine exercises (Life Fitness, Rosemont, IL) or a waitlist control group. The resistance exercise protocol included three upper body exercises: chest press, shoulder press, and pulldown, and four lower body exercises: leg press, leg curl, leg extension, and plate‐loaded leg press.

Subjects performed one set of each exercise to volitional fatigue/failure (i.e., inability to complete another repetition) with the goal of performing 8–12 repetitions at a slow and controlled pace. The latter is consistent with current recommendations for RET in older adults (Garber et al. 2011; Williams et al. 2007). All participants were asked to maintain their habitual dietary intake and physical activity level during the intervention period. Compliance to this request was confirmed using 24‐h dietary recalls and accelerometry as described below. Muscle biopsies were obtained at baseline and 48 h after the last resistance exercise session at 3 and 12 weeks of the intervention. The waitlist control group remained sedentary for a 12‐week period and had muscle biopsies at the same intervals. The control group was offered the resistance exercise intervention after completion of their participation in the study.

### Strength assessment

Upper body and leg strength were assessed at baseline and week 12 using a three‐repetition max (3‐RM) test for chest press and leg press. The 3‐RM was defined as the maximal resistance that could be moved through the full range of motion for three repetitions. Subjects began with a warm‐up set of three repetitions and resistance was progressively increased in subsequent sets to a point where the subjects could not perform three repetitions.

### Procedures

Body weight was measured to the nearest 0.1 kg using a digital scale with participants wearing light street clothing and no shoes (Scale‐Tronix model 5002, Wheaton, IL). Height was measured in cm without shoes using a wall‐mounted stadiometer. Waist circumference was measured to the nearest 0.5 cm at the umbilicus using a Gulick tape measure (Gulick, Country Technology, Inc, Gays Mill, WI). Body composition was measured via dual energy X‐ray absorptiometry (GE Lunar Prodigy Advance, software version 8.10e, GE Healthcare, Madison, WI). Skeletal muscle mass index (Fielding et al. 2011) was calculated as described previously (Fielding et al. 2011). Participants wore an accelerometer (GT1M, ActiGraph, LLC, Pensacola, FL) for a 4‐day period at baseline and again at 12 weeks to determine physical activity level outside of the training protocol measured in steps per day. Subjects completed three 24‐h dietary recalls during the week of initial and follow‐up testing. These were completed by a trained diet technician and included one weekend day and 2 weekdays. The dietary recalls were analyzed using NDS‐R version 2011 (University of Minnesota, Minneapolis MN).

Biopsies were obtained in the fasted state from the vastus lateralis muscle using a modified Bergström‐type needle (Cadence, Staunton, VA) using suction (Marinik et al. 2013) at baseline, week 3, and week 12 for both the intervention and control groups. Samples were immediately placed in SET buffer (0.25 mol/L Sucrose, 1 mmol/L EDTA, 0.01 mol/L Tris‐HCl and 2 mmol/L ATP) and stored on ice until analysis within 30 min. Muscle used for mitochondrial isolation was placed in isolation buffer (100 mmol/L Kcl, 50 mmol/L MOPS. 1 mmol/L EDTA, 5 mmol/L MgCl2, 1 mmol/L ATP, pH 7.5; all from Sigma‐Aldrich, St. Louis, MO) and stored on ice until isolation. Muscle used for quantitative real‐time polymerase chain reaction (qRT‐PCR) was placed in Trizol (Invitrogen, Carlsbad, CA) and snap‐frozen in liquid nitrogen.

Pyruvate and palmitate oxidation rates were measured in the fresh muscle homogenates by counting the ^14^CO_2_ produced from [U‐^14^C] pyruvate and [1‐^14^C] palmitic acid during incubation, respectively, as previously described (Hulver et al. 2003). The activity of CS, a biochemical marker of mitochondrial density and oxidative capacity (Blomstrand et al. 1997; Larsen et al. 2012) and *β*HAD, a key regulatory enzyme in the beta oxidation of fatty acids to acetyl Co A, were determined spectrophotometrically in muscle homogenates as described previously (Heilbronn et al. 2005; Frisard et al. 2010).

RNA was extracted using an RNeasy Mini Kit with DNase I treatment (Qiagen, Valencia, CA), according to the manufacturer's instructions. qRT‐PCR was performed using an ABI PRISM 7900 Sequence Detection System instrument and TaqMan Universal PCR Master Mix used according to the manufacturer's specifications (Applied Biosystems, Foster City, CA). Gene expression of key transcriptional regulators of mitochondrial biogenesis (peroxisome proliferator‐activated receptor‐gamma coactivator‐1 alpha [PGC‐1*α*] and transcription factor A, mitochondrial [TFAM]) and metabolism (peroxisome proliferator‐activated receptor delta) [PPAR*δ*]) in skeletal muscle were normalized to cyclophilin B mRNA levels. Primers and 5# FAM‐labeled TaqMan probes were purchased as previously validated assays (Applied Biosystems). Relative quantification of target genes were calculated using the 2^−ΔΔC^_T_ method. Derivation of the 2^−ΔΔC^_T_ equation has been described in Applied Biosystems User Bulletin no. 2 (P/N 4303859). All samples were run in triplicate.

Mitochondria were isolated and prepared as previously described with modifications (Bharadwaj et al. 2015). Briefly, the biopsy sample was homogenized using a dounce homogenizer and mitochondria were isolated using differential centrifugation. Mitochondrial protein concentrations were determined spectrophotometrically using the bicinchoninic acid assay (ThermoScientific, Pittsburg, PA).

Reactive oxygen species production at complex 1, complex III, and reverse flow of electrons in isolated mitochondria was measured using an Amplex Red Hydrogen Peroxide/Peroxidase assay kits as previously described (Mcmillan et al. 2015). All experiments were performed at 37°C. Measures for ROS levels were conducted on a microplate reader (Biotek synergy 2, Winooski, VT). Fluorescence of Amplex Red was measured using a 530 nm excitation filter and a 560 nm emission filter.

### Statistical analysis

Mann–Whitney *U* tests were performed to compare subject characteristics and changes in dependent variables between groups. Repeated measures analysis of variance was used to assess the effect of treatment (resistance exercise vs. control), time, and treatment by time interaction on the dependent variables of interest. Because we have a small sample, these analyses were rerun with standard error estimates based on 1000 bootstrap samples to obtain bias‐corrected confidence intervals by repeated re‐estimations of the parameter estimates. There were no significant differences in any of the dependent variables between statin users and nonusers at baseline or following the resistance exercise intervention. In addition, the exclusion of statin‐users from the analysis did not alter the conclusions of the study. As such, only the pooled data are presented. All data are presented as means ± standard deviation. Significance level was set a prior at *P* < 0.05.

## Results

Subject characteristics are shown in Table 1. There were no significant differences in body weight or body composition variables at baseline in the waitlist and resistance exercise group. However, body mass index was lower (*P* < 0.05) in the resistance exercise group compared with the waitlist control group. Body weight, body fat, fat‐free mass, or skeletal muscle mass index did not change (all *P* > 0.05) following the intervention in either group. Waist circumferences was similar (*P* > 0.05) in the two groups at baseline. However, there was a reduction in waist circumference following RET (97.4 ± 8.7–95.3 ± 8.2 cm, *P* < 0.05) that was not evident in the waitlist group (100.5 ± 4.6– 100.9 ± 4.1 cm, *P* > 0.05).

**Table 1:** Baseline and 12‐week subject characteristics of the waitlist control and resistance exercise groups

	Waitlist group, *n* = 8		Resistance exercise group, *n* = 11		*P*‐value (group)	*P* ‐value (time)	*P* ‐value (group × time)
	Baseline	12‐weeks	Baseline	12‐weeks			
Age, years	67.9 ± 5.8	—	64.8 ± 4.1	—	0.18	—	—
Weight, kg	85.1 ± 7.4	84.6 ± 6.9	80.1 ± 10.1	80.4 ± 9.9	0.21	0.97	0.30
BMI, kg/m2	27.8 ± 2.0	27.8 ± 2.1	25.8 ± 2.6	26.0 ± 2.4	0.05	0.79	0.36
Body fat, %	28.3 ± 3.2	28.0 ± 3.4	26.2 ± 7.0	25.64 ± 6.0	0.28	0.24	0.89
Fat‐free mass, kg	57.4 ± 5.8	57.5 ± 6.0	55.1 ± 5.1	55.8 ± 5.3	0.46	0.07	0.43
Skeletal muscle index, ALM/m^2^	9.79 ± 0.83	10.0 ± 0.9	9.6 ± 0.6	9.8 ± 0.6	0.55	˂0.01	0.87

Values are means ± S.D

BMI, Body Mass Index; ALM, Appendicular Lean Mass.

The participants in the resistance exercise group completed 31.8 ± 2.2 (88.7 ± 6.4%) of the 36 training sessions. As expected, chest press and leg press 3RM each increased ~34% (both *P* < 0.01), respectively, following 12 weeks of RET. Leg press 3 RM increased from 110 ± 28 to 148 ± 23 kg (*P* < 0.05) with RET; there was no significant change observed in the waitlist control group (122 ± 30–115 ± 26 kg). Chest press 3 RM increased from 52 ± 13 to 70 ± 17 kg (*P* < 0.05) with RET. However, chest press 3 RM was unchanged (*P* > 0.05) in the waitlist control group after 12 weeks (baseline: 49 ± 15 vs. 12 weeks: 48 ± 15 kg). There was no significant difference in habitual physical activity or total calorie and macronutrient intake at baseline in the two groups and no change in these variables following the intervention (data not shown).

There were no significant differences in any measure of substrate oxidation between the two groups at baseline. Pyruvate oxidation did not change (*P* > 0.05) following the intervention (Table 2). Similarly, there were no significant changes in complete (CO_2_ produced), incomplete [acid soluble metabolites (ASM)], or total fatty acid oxidation [CO_2_ produced plus ASM (FAO)], or the ratio of total FAO:ASM following the intervention (all *P* > 0.05). TFAM, PGC‐1*α*, or PPAR *δ* mRNA were similar at baseline in the two groups and there were no significant changes in the expression of these transcripts in either group following the intervention (all *P* < 0.05) (Table 3). CS activity was lower (*P* < 0.05) in the waitlist compared with resistance exercise group at baseline. However, there was no significant change in CS activity in either group following the intervention (*P* > 0.05) (Table 3). *β*‐HAD activity was similar (*P* > 0.05) in the two groups at baseline and there was a ~30% increase (*P* < 0.05) in *β*‐HAD activity with RET at 12 weeks (Table 3). No significant change in *β*‐HAD activity was observed following 12 weeks in the waitlist control group. Mitochondrial ROS production at complex I, complex III, and in reverse flow of electrons were similar (*P* > 0.05) in the waitlist and resistance exercise groups at baseline but there were no significant changes in any of these variables in either group following the intervention (all *P* > 0.05) (Table 4).

**Table 2:** In vivo skeletal muscle substrate oxidation before, during, and following the intervention

	Waitlist group, *n* = 8			Resistance exercise group, *n* = 11			*P* ‐value (group)	*P* ‐value (time)	*P* ‐value (group × time)
	Baseline	3‐weeks	12‐weeks	Baseline	3‐weeks	12‐weeks			
PDH (C02 produced) [(nmol mg/protein)/h]	398.4 ± 202.1	400.2 ± 291.5	442.7 ± 194.2	420.6 ± 179.0	352.9 ± 109.3	570.7 ± 271.7	0.60	0.06	0.26
FAO (C0_2_ produced) [(nmol mg/protein)/h]	1.0 ± 0.7	1.0 ± 1.0	1.0 ± 0.8	1.1 ± 0.4	0.8 ± 0.3	0.8 ± 0.4	0.71	0.80	0.31
Total FAO [(nmol mg/protein)/h]	14.2 ± 5.4	14.4 ± 10.5	14.9 ± 5.2	12.5 ± 5.0	12.1 ± 3.3	13.8 ± 4.2	0.35	0.41	0.76
FAO (ASM) [(nmol mg/protein)/h]	13.2 ± 4.8	13.4 ± 9.9	13.8 ± 3.9	11.4 ± 4.6	11.2 ± 3.4	12.8 ± 4.0	0.33	0.40	0.68
FAO (C02:ASM)	0.1 ± 0.03	0.1 ± 0.04	0.1 ± 0.04	0.1 ± 0.1	0.1 ± 0.1	0.1 ± 0.04	0.26	0.26	0.12

Values are means ± S.D

PDH (CO2), Pyruvate dehydrogenase (Carbon dioxide produced); FAO (CO2 produced), Fatty acid oxidation (Carbon dioxide produced); Total FAO, Total fatty acid oxidation (united needed?); FAO (ASM), Fatty acid oxidation (Acid soluble metabolites); FAO (CO2:ASM), Fatty acid oxidation (Carbon dioxide:Acid soluble metabolites; BHAD, Beta hydroxacyl coenzyme A dehydrogenase; CS, Citrate synthase.

**Table 3:** Mitochondrial target gene expression and oxidative enzyme activity before, during, and following the intervention

	Waitlist Group, *n* = 8			Resistance Exercise Group, *n* = 11			*P* ‐value (group)	*P* ‐value (time)	*P* ‐value (group x time)
	Baseline	3‐weeks	12‐weeks	Baseline	3‐weeks	12‐weeks			
Tfam mRNA	1.03 ± 0.66	0.75 ± 0.45	2.08 ± 6.51	2.32 ± 3.95	1.96 ± 2.75	1.48 ± 6.51	0.74	0.40	0.20
PGC‐1*α* alpha mRNA	1.06 ± 0.46	1.54 ± 0.74	0.94 ± 0.26	1.12 ± 0.37	1.28 ± 0.46	1.01 ± 0.68	0.65	0.09	0.94
PPARδ mRNA	1.26 ± 0.70	0.85 ± 0.29	0.94 ± 0.71	0.98 ± 0.24	0.99 ± 0.23	0.83 ± 0.37	0.65	0.14	0.94
BHAD activity (*μ*mol/mg protein/min)	24.1 ± 5.8	22.4 ± 5.5	23.8 ± 6.7	24.4 ± 7.4	26.7 ± 5.1	31.3 ± 4.7	0.07	0.03	0.05
CS activity (*μ*mol/mg protein/min)	59.9 ± 18.3	62.7 ± 11.6	55.0 ± 12.1	74.8 ± 15.5	83.0 ± 30.7	74.4 ± 17.6	0.00	0.43	0.64

Tfam, Transcription factor A; mitochondria; PGC‐1*α,* Peroxisome proliferator‐activated receptor‐*γ* co‐activator alpha; *PPARδ,* peroxisome proliferator‐activated receptor delta.

Values are means ± S.D.

**Table 4:** Mitochondrial reactive oxygen species production before, during and following the intervention

	Waitlist Group, *n* = 8			Resistance Exercise Group, *n* = 11			*P* ‐value (group)	*P* ‐value (time)	*P* ‐value (group x time)
	Baseline	3‐weeks	12‐weeks	Baseline	3‐weeks	12‐weeks			
ROS‐ complex I (au)	202.8 ± 68.9	131.1 ± 24.7	185.2 ± 84.8	113.0 ± 25.7	135.2 ± 37.4	125.1 ± 29.9	0.22	0.57	0.53
ROS‐ complex III (au)	192.9 ± 72.3	128.1 ± 41.7	191.5 ± 55.2	136.7 ± 38.0	167.8 ± 83.8	151.4 ± 57.8	0.39	0.37	0.18
ROS‐ Reverse flow electrons	823.3 ± 290.6	589.1 ± 122.2	666.6 ± 139.6	608.6 ± 231.2	597.8 ± 290.0	689.6 ± 213.0	0.27	0.44	0.10

ROS, Reactive oxygen species.

Values are means ± S.D.

## Discussion

The major new finding from this study is that low volume RET did not increase in vitro skeletal muscle oxidative capacity. The latter is based on three important observations. First, gene expression of several key transcriptional regulators of mitochondrial function and metabolism did not change following the intervention. Second, neither skeletal muscle fatty acid oxidation nor pyruvate oxidation increased following RET in older adults in this study. Finally, CS activity, a commonly used marker of mitochondrial density and oxidative capacity, was not altered following the completion of 12 weeks of RET.

To our knowledge, our study is the first to determine the impact of RET on in vitro skeletal muscle substrate oxidation. In addition, we included measurements of gene expression of key transcriptional regulators of mitochondrial biogenesis and metabolism and enzymatic activity reflective of oxidative capacity of skeletal muscle (i.e., CS activity) to provide a comprehensive assessment of skeletal muscle oxidative capacity following RET. The lack of change in CS activity in this study is consistent with previous study in older adults (Parise et al. 2005a,b) and a minority of previous studies in young adults (see (Tang et al. 2006) for summary). Although *β*HAD activity did increase significantly following the completion of 12 weeks of RT this was not associated with an obvious increase in in vitro fatty acid oxidation in this study.

Our findings, albeit based on only a few gene transcripts, are inconsistent with global enrichment of genes associated with mitochondrial function in skeletal muscle following RET in older adults reported by Melov et al. (2007). However, an important limitation of the latter study was that no functional measurements of mitochondrial function or substrate oxidation were included. As such, it is important to emphasize that making any conclusions based solely on gene expression (or enzyme activity) without the inclusion of functional measurements is tenuous.

Mitochondrial ROS production was not reduced following 12 weeks of RET in older adults in this study. These observations appear inconsistent with the prior observations suggesting that lipid peroxidation was reduced (Vincent et al. 2002) and oxidative damage to DNA were reduced (Parise et al. 2005a) and antioxidant enzyme activity was increased (Parise et al. 2005a,b) following RET in older adults. Future studies involving several measures of oxidative stress (e.g., ROS production, antioxidant activity, and oxidative damage to DNA) will be necessary to clarify this issue.

There are some limitations in this study that should be considered. First, our sample size was small and included only healthy, older males. We cannot rule out the possibility that RET would increase skeletal muscle oxidative capacity in less healthy or frail older adults.

Second, it is possible that the inclusion of statin‐treated individuals in this study may have impaired our ability to detect improvements in skeletal muscle oxidative capacity and reduction mitochondrial ROS production following RET (Murlasits and Radak 2014). There were no significant differences in the changes in skeletal muscle oxidative capacity or mitochondrial ROS production in the statin users and non‐users following RET in this study. However, the sample size of the two groups were small. As such, we cannot exclude the possibility that statin use impaired our ability to detect changes in skeletal muscle substrate and mitochondrial ROS production in this study. Future studies with larger sample sizes in participants not taking statins will be necessary to confirm our findings.

Finally, we did not quantify muscle hypertrophy following RET in this study. As such, we cannot exclude the possibility that the lack of change in skeletal muscle oxidative capacity was the result, at least in part, to a “dilution” of mitochondria in hypertrophied muscle. However, we did not observe a change in skeletal muscle oxidative capacity following 3 weeks of RET when little or no obvious hypertrophy is expected (Sale 1988). In addition, Tang et al. (2006) reported increase in CS and BHAD activities following 12 weeks of resistance exercise that produced significant muscle hypertrophy in young men. As such, we do not believe a “dilution” effect is responsible for our observations.

Our study may have important implications for the design of exercise programs for older adults. It is important to emphasize that the design of such programs should consider individual preference and the desired outcome. For example, reductions in strength and muscle mass with advancing age are associated with disability, loss of independence, and mortality (Berger and Doherty 2010). As such, RET has become increasingly important priority for older adults particularly given the changing demographic of the aging population. However, mitochondrial dysfunction and ROS production have been implicated in apoptosis, muscle loss, and sarcopenia (Cadenas and Davies 2000; Joseph et al. 2015). As such, some amount of large muscle, dynamic exercise may need to be included with RET to maximize skeletal muscle adaptations in older adults. Nevertheless, future studies are necessary to clarify the importance of different modes and amounts/intensities of exercise on skeletal muscle oxidative capacity in older adults.

In summary, the results of this study indicate that a low volume resistance exercise intervention does not increase skeletal muscle oxidative capacity or reduce mitochondrial ROS production in older adults. Importantly, resistance training alone may be inadequate to reverse skeletal muscle aging and some large muscle, dynamic exercise may be needed for optimal skeletal muscle adaptations in older adults. This possibility should be considered when designing exercise programs for middle‐aged and older adults.

## Conflict of Interest

None declared.
